# The Yin-Yang Regulation of Reactive Oxygen Species and MicroRNAs in Cancer

**DOI:** 10.3390/ijms20215335

**Published:** 2019-10-26

**Authors:** Kamesh R. Babu, Yvonne Tay

**Affiliations:** 1 Cancer Science Institute of Singapore, National University of Singapore, Singapore 117599, Singapore; csirbk@nus.edu.sg; 2 Department of Biochemistry, Yong Loo Lin School of Medicine, National University of Singapore, Singapore 117597, Singapore

**Keywords:** ROS, oxidative stress, antioxidants, miRNA, cancer

## Abstract

Reactive oxygen species (ROS) are highly reactive oxygen-containing chemical species formed as a by-product of normal aerobic respiration and also from a number of other cellular enzymatic reactions. ROS function as key mediators of cellular signaling pathways involved in proliferation, survival, apoptosis, and immune response. However, elevated and sustained ROS production promotes tumor initiation by inducing DNA damage or mutation and activates oncogenic signaling pathways to promote cancer progression. Recent studies have shown that ROS can facilitate carcinogenesis by controlling microRNA (miRNA) expression through regulating miRNA biogenesis, transcription, and epigenetic modifications. Likewise, miRNAs have been shown to control cellular ROS homeostasis by regulating the expression of proteins involved in ROS production and elimination. In this review, we summarized the significance of ROS in cancer initiation, progression, and the regulatory crosstalk between ROS and miRNAs in cancer.

## 1. Introduction

Reactive oxygen species (ROS) are free radicals, ions, or molecules with a single unpaired electron. ROS, including hydrogen peroxide (H_2_O_2_), hydroxyl radicals (OH^−^), nitric oxide (NO), and superoxide radicals (O_2_^−^) are highly reactive and generated as a byproduct during metabolic processes in various subcellular compartments of a cell [1]. Mitochondria are the main cellular source of ROS. However, ROS are also generated in other cellular organelles including endoplasmic reticulum, lysosomes, and peroxisomes [2]. At lower concentrations, ROS play significant roles in various physiological functions including gene activation, cell growth, proliferation, survival, apoptosis, chemical reaction modulation, blood pressure control, prostaglandin biosynthesis, embryonic development, cognitive function, and immune response [3,4]. However, at higher concentrations, ROS can cause oxidative damage via oxidation of macromolecules such as DNA, RNA, proteins, and lipids that can contribute to the pathogenesis of various diseases including cancer [5,6,7,8,9]. Elevated ROS production is associated with tumorigenesis and suggested to be a hallmark of cancer. Nevertheless, the molecular mechanisms responsible for sustained high ROS levels in cancer is not well understood.

MicroRNAs (miRNAs) are a class of small non-coding RNAs that are approximately 22 nucleotides long and regulate gene expression at the post-transcriptional level [10]. They regulate gene expression by binding to the target messenger RNA (mRNA) transcript which activates either degradation or translation suppression based on the extent of basepairing. However, several studies reported that miRNAs can also target and regulate the stability of non-coding RNAs. Studies have demonstrated that the deregulation of miRNA expression is associated with cancer development, and miRNAs may function as potential oncogenes or tumor suppressors [11]. Surprisingly, studies show the existence of a regulatory connection between ROS and miRNA. For example, H_2_O_2_ treatment has been shown to dysregulate the expression of certain miRNAs in vascular smooth muscle cells and macrophage cells [12,13]. Another study has shown that miR-30e regulates oxidative stress and ROS levels by targeting SNAI1 mRNA in human umbilical endothelial vein cells [14]. These findings suggest that ROS and miRNAs may co-regulate each other in cancer to maintain cellular ROS levels that support cancer development. In this review, we discuss the significance of ROS in cancer development, as well as the crosstalk between ROS and miRNAs in the regulation of redox homeostasis and cancer progression.

## 2. Significance of ROS in Cancer Development 

ROS are required by cells to carry out physiological cellular functions and this is also true in the case of cancer cells. However, cancer cells show elevated levels of ROS when compared to normal cells, which is mainly due to persistent and high metabolic rate in mitochondria, endoplasmic reticulum (ER). and cell membranes. In this section, we discuss how ROS play a significant role in the whole process of cancer development, including initiation, promotion, and progression. 

### 2.1. ROS in Cancer Initiation

ROS are potent mutagens that can stimulate cancer initiation. High levels of ROS oxidize DNA bases resulting in DNA lesions including base damage, strand breaks, and mutations, which are usually repaired by the endogenous DNA repair enzymes of the base excision repair, nuclear excision repair, or mismatch repair pathways [15]. Cells unable to repair DNA lesions undergo apoptosis to prevent the passage of DNA mutations to progeny cells. However, under certain conditions, cells harboring DNA lesions evade apoptosis, which eventually leads to cancer. In a similar fashion to DNA, RNA also undergoes oxidation under oxidative stress that results in strand breaks and oxidative base modifications. Oxidized mRNA can cause several defects during protein translation, which include synthesis of truncated, mutated, or non-functional proteins, ribosome stalling, and ribosome dysfunction [6]. Oxidized RNA can promote the pathogenesis of chronic degenerative diseases including cancer [7]. For example, oxidation of tumor suppressor mRNAs results in the synthesis of mutated or truncated proteins that lack proper function, and this may lead to carcinogenesis. It is important to note that RNA oxidation is not limited only to mRNA as all RNA species including non-coding RNAs are subjected to oxidative damage. Since several studies have shown the significant participation of non-coding RNAs, including miRNAs and long non-coding RNAs (lncRNAs) in cancer development [16], oxidative modification of non-coding RNAs may also promote cancer initiation. ROS-induced mutation or modification is not only restricted to nucleotides, but even protein molecules are also susceptible to such modifications. Oxidation of proteins by ROS results in amino acid modification, protein carbonylation, nitration of tyrosine and phenylalanine residues, protein degradation, or formation of cross-linked proteins or glycated proteins [17,18]. Oxidized amino acid residues can affect their protein activity. For example, oxidation of DNA polymerase affects its fidelity during replication/synthesis, transcription, or DNA repair activity, which is closely associated with cancer initiation [19]. Finally, ROS can also damage polyunsaturated or polydesaturated fatty acids by the process of lipid peroxidation which generates various toxic molecules including malondialdehyde, 2-alkenals, 4-hydroxynonenal (HNE), and lipoperoxyl radical (LOO^−^) [9,20]. The LOO^−^ reacts with the lipids to generate lipid peroxides, which are unstable and can produce new peroxyl and alkoxy radicals. These radicals may further increase the oxidation of macromolecules. Furthermore, HNE is a chemically reactive molecule that can react with macromolecules and form covalent modifications, which has been proposed as the mechanism to induce carcinogenesis [20]. These studies indicate that higher levels of ROS are detrimental to cells and can increase the risk of developing cancer. 

### 2.2. ROS in Cancer Cell Proliferation

ROS function as secondary messengers in cellular signaling and activate ROS-sensitive signaling pathways by regulating protein activity through the reversible oxidation of target proteins. Redox-sensitive signaling pathways, including the mitogen-activated protein kinase (MAPK)/extracellular signal-regulated kinase (ERK), phosphoinositide-3 kinase (PI3K)/protein kinase B (AKT), and nuclear factor κ-B (NF-κB) signaling pathways, are constantly upregulated in various cancer subtypes, where they play a pivotal role in cell proliferation, growth, protein synthesis, glucose metabolism, cell survival, and inflammation [21]. Activation of MAPK/ERK signaling has been shown to increase anchorage-independent growth, cell survival, and motility of many cancer subtypes including breast cancer, leukemia, melanoma, and ovarian cancer. Studies have shown that high ROS levels in cancer cells can elevate MAPK/ERK signaling and can increase cancer cell proliferation [19]. Analogously, high levels of ROS, either produced endogenously or added exogenously, have shown to increase the activation PI3K/AKT signaling pathway in breast and ovarian cancer. Furthermore, studies show that elevated ROS levels can activate the transcription factor NF-κB. Oxidative stress-induced through the exogenous treatment of sodium arsenite, rotenone, H_2_O_2_, or through inhibition of endogenous antioxidants elevated the NF-κB activation and increased cancer cell proliferation [19,22]. Moreover, ROS play a significant role in the cell cycle by regulating mRNA levels of cyclins that promote G1 to S phase transition, which include cyclin B2, cyclin D3, cyclin E1, and cyclin E3 [23]. In breast cancer cells, ROS generated by sodium arsenite treatment promote S phase transition and aberrant cell proliferation [24], whereas reduction of ROS levels through antioxidant N-acetyl cysteine (NAC) treatment reduces cyclin D1 levels and slowed the G1 to S phase transition in the non-cancerous human breast epithelial cells [25]. All these studies suggest that besides being a highly reactive mutagen, ROS can also function as a secondary messenger that mediate physiological signaling pathways involved in cell proliferation, thus higher ROS production in cancer cells favor cancer progression through elevated and sustained activation of these pathways. 

### 2.3. ROS in Cancer Metastasis

Metastasis is a multistep process that involves the spread of cancer cells from its original site to distal parts of the body, the process comprises migration, invasion, intravasation into the blood, anchorage-independent survival in the blood, and extravasation into distal organs [26]. Several studies show that ROS levels are increased in cells that undergo metastasis, and they play a significant role in the cancer cell metastasis. A study has shown that endogenous ROS levels are increased in circulating melanoma cells and metastasis nodules of xenografted mice compared to primary subcutaneous tumors [27]. Importantly, cancer cells treated with H_2_O_2_ have shown high metastasis upon injected intravenously into mice. Likewise, a sub-population of the breast cancer cells that has elevated intracellular ROS levels compared to the parental cells exhibits high motility and metastasized to distant organs including lung, liver, and spleen [19]. It is noteworthy that the levels and activity of endogenous antioxidants are decreased in metastatic cancer cells. For example, the levels and catalytic activity of manganese-dependent superoxide dismutase (MnSOD) are lower in highly invasive pancreatic cancer cells and metastatic breast cancer cells [28,29]. Cancer cells go through epithelial to mesenchymal transition (EMT) before migrating to distant sites of the body. During the EMT process, expression of matrix metalloproteinases (MMPs) is increased to mediate degradation and reorganization of extracellular matrix and their elevated activation is associated with tumor growth, angiogenesis, invasion, and metastasis [30]. ROS play a significant role in the EMT process in which they regulate the expression of MMPs and their inhibitors tissue inhibitor of metalloproteinases (TIMP) [31]. A study has shown that treatment of MMP-3, a stromal protease whose expression is upregulated in mammary tumors, has increased cellular ROS and induced EMT in murine mammary epithelial cells. In contrast, scavenging cellular ROS through NAC treatment abrogated MMP-3-induced EMT, suggesting that high levels of cellular ROS can lead to malignant transformation [32]. Moreover, ROS also facilitate metastasis by increasing vascular permeability through various mechanisms. Oxidative stress in endothelial cells mediate Rac-1-induced loss of cell–cell adhesion and loosens the endothelium integrity, which favors the cancer cell intravasation [33]. ROS regulate the expression of IL-8 and intracellular adhesion protein 1 (ICAM-1) via NF-κB activation. Both IL-8 and ICAM-1 regulate transendothelial migration of tumor cells [34,35]. Furthermore, ROS induce actin reorganization in vascular endothelial cells through p38-mediated phosphorylation of the heat shock protein Hsp27, which may contribute to promote invasive processes [36]. Taken altogether, these studies suggest that ROS has a versatile role in the pathogenesis of cancer, therefore it would be interesting to identify further novel roles of ROS in other physiological processes that could possibly support the process of cancer development.

### 2.4. ROS in Cancer Stem Cells

Cancer stem cells (CSCs) are a subset of tumorigenic cells that possess similar characteristics as normal stem cells, in particular the capabilities of self-renewal or differentiation. Interestingly, CSCs have been shown to have a high capacity to grow into tumors. Similar to cancer cells, ROS also play an important role in CSCs. However, in contrast to cancer cells in which ROS levels are elevated, CSCs exhibit lower levels of ROS. This is similar to the levels found in normal stem cells [37]. The lower cellular ROS levels in CSCs are associated with increased expression of ROS scavenging systems and are essential for the maintenance of self-renewal and stemness. A study has shown that pharmacological depletion of ROS scavengers in breast CSCs reduces their clonogenicity and results in radiosensitization [38]. Conversely, ovarian CSCs exhibit higher mitochondrial ROS production, and inhibition of the mitochondrial respiratory chain in CSCs results in apoptosis [39]. Furthermore, a study has shown that the population of hematopoietic stem cells (HSCs) with higher ROS levels possess higher myeloid differentiation potential compared to the HSC fraction with lower ROS levels [40]. These findings suggest that ROS levels in CSCs are crucial for their survival and differentiation. Nevertheless, the effects of ROS and the regulation of ROS levels in CSCs have not been studied extensively. Future investigations may unravel the molecular mechanisms behind the regulation of redox homeostasis in CSCs. 

## 3. ROS Regulate MiRNA Expression

Accumulating studies show functional regulatory links between ROS and miRNAs in carcinogenesis. ROS also contribute to cancer development by regulating the expression of miRNAs that target genes responsible for enhancing or suppressing carcinogenesis. In this section, we discuss how the ROS affect the miRNA expression in cancer via different mechanisms including alteration of epigenetic signatures, transcription, and biogenesis.

### 3.1. Regulation of MiRNA Expression via Epigenetic Modifications

**Figure 1:**
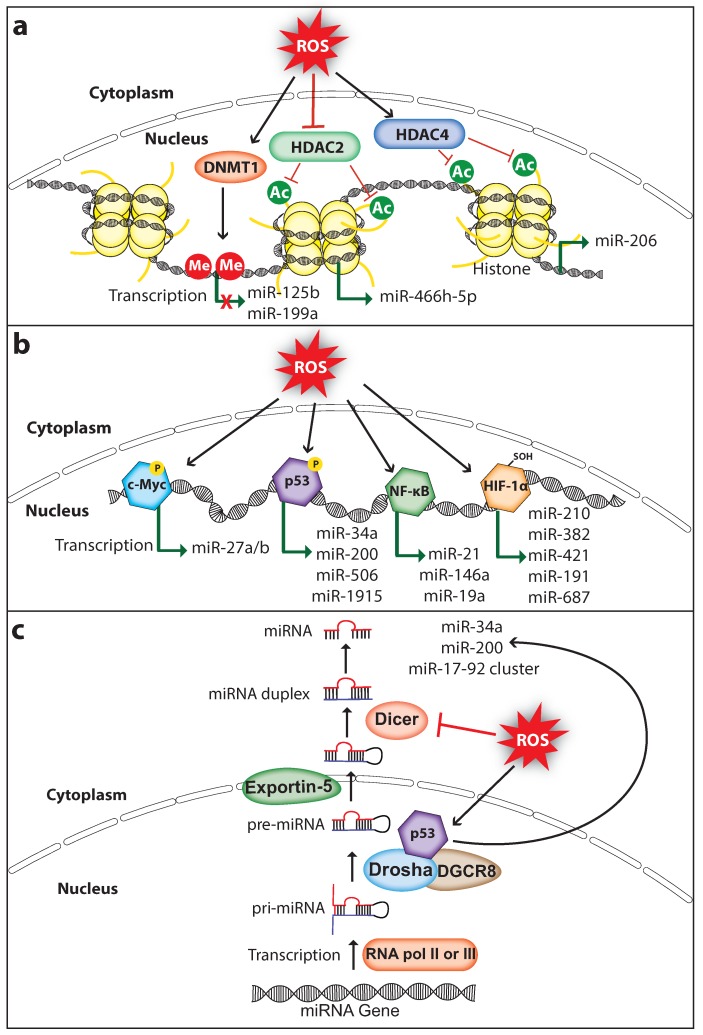
ROS regulate miRNA expression and processing in cancer. (**a**) ROS induce epigenetic modifications to regulate miRNA expression. ROS affect DNMT1 (DNA methyltransferase 1), HDAC2 (Histone deacetylase 2), or HDAC4 (Histone deacetylase 4) to either inhibit or activate miRNA expression. (**b**) ROS can induce miRNA transcription through activating transcription factors c-Myc, p53, NF-κB (nuclear factor κ-B), or HIF-1α (hypoxia-inducible factor 1-alpha). (**c**) ROS affect miRNA biogenesis and maturation through regulating the activity and expression of miRNA processing enzymes Drosha and Dicer, respectively. Me, methyl group; Ac, acetyl group; P, phosphoryl group (The black arrow indicates upmodulation, the red T arrow indicates inhibition, the green arrow indicates transcription activation, the green arrow with red cross indicates transcription inhibition).

Dysregulated miRNA expression in cancer is associated with altered DNA methylation and histone modifications such as acetylation, methylation, and phosphorylation. ROS can regulate miRNA expression by altering the epigenetic signatures including DNA methylation or histone modifications (Figure 1a). For example, ROS inhibit the expression of miR-199a and miR-125b in ovarian cancer cells via increasing promoter methylation of the miR-199a and miR-125b genes, which is mediated by the DNA methyltransferase 1 (DNMT1) [41]. Interestingly, overexpression of miR-199a and miR-125b in ovarian cancer cells decreased the expression of the hypoxia-inducible factor 1-alpha (HIF-1α) and vascular endothelial growth factor, which suppressed tumor-induced angiogenesis [42]. Histone modifications play an important role in chromatin remodeling in order to regulate gene transcription. Histone acetylation is a type of histone modification in which the lysine residues of histone are acetylated to relax the chromatin structure for gene transcription. In contrast, deacetylation of lysine residues catalyzed by the histone deacetylases (HDACs) causes chromatin condensation and transcriptional gene silencing [43]. ROS can regulate the activity of HDACs. For example, the Cys667 and Cys669 amino acid residues of HDAC4 are oxidized to form an intramolecular dis-sulfide bond, which promotes its nuclear export [44]. Cancer cells promote nuclear translocation of HDAC4 by increasing endogenous antioxidants, which decreases miR-206 expression through deacetylation of its promoter and promotes cancer progression [45,46]. Furthermore, oxidative stress-induced by glucose depletion increases the expression of miR-466h-5p by inhibiting HDAC2 activity, which results in increased apoptosis due to the fact that miR-466h-5p directly targets and downregulates many anti-apoptotic proteins including BCL212, DAD1, BIRC6, STAT5A, and SMO [47,48]. These findings suggest that ROS can affect the epigenetic status of miRNA genes thereby regulating its expression in cancer. It is important to note that ROS-mediated regulation of DNMT1 and HDACs in cancer may change its global epigenetic signature, therefore the expression of other genes including oncogenes and tumor suppressors can also be activated or silenced. 

### 3.2. Regulation of MiRNA Expression via Transcription Factors

ROS can also control miRNA expression by regulating the ROS-responsive transcription factors that activate miRNA transcription (Figure 1b). ROS regulate the activation of transcription factors through direct or indirect mechanisms. The activated transcription factor binds to the target miRNA promoter and upregulates miRNA transcription. 

#### 3.2.1. C-Myc

C-Myc is a well-studied transcription factor and characterized as an oncoprotein whose expression is elevated in a wide range of tumors. It promotes tumorigenesis by activating the transcription of several oncogenes including the miR-17-92 cluster, or by inhibiting the transcription of tumor-suppressors including let-7a which functions as a negative regulator of CSC features by regulating PTEN and Lin28b expression in pancreatic and prostate cancer [49]. c-Myc is a redox-sensitive transcription factor. Under oxidative stress, ROS cause ERK-dependent phosphorylation at the Ser62 amino acid residue of c-Myc which enhances the c-Myc recruitment to the promoter of gamma-glutamyl-cysteine synthetase, the rate-limiting enzyme catalyzing glutathione (GSH) synthesis. The c-Myc phosphorylation-dependent activation of GSH promotes the survival of cancer cells under oxidative-stress conditions [50]. Lithocholic acid (LCA)-induced ROS increased c-Myc expression in the human hepatocellular carcinoma (HCC) cells and in mouse liver. Importantly, LCA mediated c-Myc overexpression activates the expression of miR-27a/b that promotes HCC proliferation [51]. miR-27a/b directly targets and suppresses the expression of nuclear factor-erythroid 2-related factor 2 (NRF2) and prohibitin 1 (PHB1), a mitochondrial chaperone function as a tumor suppressor in liver cancer [52], whereas knockdown of c-Myc or miR-27a/b in Huh-7 cells rescued the LCA-mediated suppression of NRF2 and PHB1. This suggests that the interplay of ROS, c-Myc, and miR-27 has a significant role in HCC progression. 

#### 3.2.2. P53

The tumor suppressor protein p53 maintains genome integrity by inducing antiproliferative programs such as cell cycle arrest, senescence, and apoptosis through differential activation of key effector genes including the tumor suppressor miR-34a [53,54]. p53 is an oxidative stress-responsive transcription factor whose expression can be induced by ROS to protect genome stability via selectively activating its target genes [55]. Furthermore, the transcriptional activity of p53 is affected by oxidative stress, as the endogenous antioxidants thioredoxin (TRX) and GSH modify the cysteine amino acids of p53, which affects p53 activity including DNA binding capacity, activation of target gene transcription, and apoptosis induction [56,57,58]. A study has shown that H_2_O_2_ treatment phosphorylates the Ser33 amino acid residue of p53 in hepatic cells, which promotes miR-200 transcription and cell death [59]. Interestingly, p53 knockdown reversed the H_2_O_2_ mediated miR-200 expression [60], confirming that miR-200 expression under oxidative stress is p53-dependent. Importantly, miR-200 has shown to function as a tumor suppressor by inhibiting the CSC self-renewal potential and EMT process in various cancer subtypes including bladder cancer, gastric cancer, ovarian cancer, pancreatic cancer, and prostate cancer [49,61,62]. Furthermore, ROS mediated p53 activation also upregulates the expression of miR-506, which inhibited the growth of lung tumor in-vitro and in-vivo [63]. In addition, expression of miR-34a-5p and miR-1915 is regulated by p53 in HCC cells during oxidative stress [64]. Moreover, miR-34 inhibits pancreatic CSC proliferation, self-renewal, and induces apoptosis and cell cycle arrest [49]. Altogether, these studies strongly suggest that p53 mediates anticancer roles through promoting the expression of tumor suppressor miRNAs in a redox-dependent fashion.

#### 3.2.3. NFκB

NF-κB is an inducible transcription factor that plays a pivotal role in DNA transcription, cytokine production, cell proliferation, survival, differentiation, cell cycle regulation, and especially in inflammation [65]. The activity of NF-κB is inhibited by its inhibitor IκB which sequesters NF-κB in the cytosol to prevent its translocation to the nucleus. The canonical NF-κB activation is mediated through the degradation of IκB, induced via site-specific phosphorylation by NF-κB-inducing kinase (NIK) and IκB kinase (IKK) protein complex, consisting of IKKα, IKKβ, and NF-κB essential modulator. ROS activate the NF-κB pathway by activating NIK through oxidative inhibition of regulatory phosphatases, and through tyrosine phosphorylation of IκBα [22]. NFκB mediates transcription of several miRNAs including let-7, miR-21, and miR-146 [66]. miR-21 is a well-studied oncomiR which mediates pro-survival and anti-proliferative effects through directly targeting and suppressing the expression of tumor suppressors such as PTEN, PDCD4, IGFBP3, and MKK3 [67,68,69,70]. Overexpression of miR-21 is associated with the progression of many cancer types and considered as a biomarker and target for cancer treatment [71]. Interestingly, miR-21 is elevated in breast CSC subpopulations and regulates the EMT phenotype [49]. ROS-induced miR-21 expression has been shown to contribute to the invasion and metastasis of prostate cancer [72]. NFκB activates miR-21 transcription by directly binding to the promoter of the miR-21 gene [73]. Likewise, ROS-activated NFκB can also upregulate miR-146a transcription, which suppresses the progression of acute myeloid leukemia (AML) [74]. In contrast, berberine-treatment-induced oxidative stress, suppressed miR-21 expression by inhibiting the nuclear translocation of NFκB in human multiple myeloma cells, which induces apoptosis [75]. In addition, oxidative stress deactivated NFκB activity that downregulated miR-19a transcription and activated apoptosis of the pheochromocytoma cells [76]. These findings suggest that the transcription factor NFκB can be either activated or inhibited under oxidative stress.

#### 3.2.4. HIF-1α 

HIF-1α is a subunit of heterodimeric transcription factor hypoxia-inducible factor 1, which regulates the expression of genes involved in the process of angiogenesis and erythropoiesis, which is important for blood vessel formation and the survival of cells under hypoxic condition [77,78]. Under hypoxia, HIF-1α activates the transcription of certain miRNAs called hypoxamiRs, which function as key regulators of the cell against decreased oxygen tension [79]. miR-210 is one such miRNA whose transcription is activated through direct binding of HIF-1α to the hypoxia-responsive element located within its promoter. Interestingly, miR-210 can negatively regulate HIF-1α expression by directly targeting its mRNA forming a negative-feedback loop, and disruption of this loop has been implicated in autoimmune diseases and tumor initiation [79,80]. Studies have shown that miR-210 promotes CSC proliferation, migration, metastasis, and self-renewal [49]. Furthermore, HIF-1α activates the transcription of many other miRNAs including miR-382, miR-421, miR-191, and miR-687 that promote migration, angiogenesis, metastasis, tumor growth, or drug resistance in cancer [81,82,83,84]. ROS regulate HIF-1α directly by oxidizing the Cys533 amino acid residue of HIF-1α, which increases the HIF-1α protein stability under oxidative stress [85]. In addition, ROS can activate HIF-1α indirectly through downregulating SIRT1 deacetylase, which results in acetylation at the Lys647 amino acid residue of HIF-1α [86]. This strongly suggests that ROS may regulate the expression of a broad range of miRNA genes in cancer by regulating the redox-sensitive HIF-1α transcription factor.

### 3.3. Regulation of MiRNA Processing

ROS can also affect miRNA expression by regulating proteins involved in miRNA processing. Generally, miRNAs are transcribed as primary miRNA (pri-miRNA) transcripts by RNA polymerase II or RNA polymerase III. Pri-miRNAs are then processed into premature miRNA (pre-miRNA) transcripts that are approximately 60–70 nucleotide long by the RNA-specific RNAse III type ribonuclease Drosha and DGCR8 protein complex. The pre-miRNA hairpins are then exported to the cytoplasm by the Exportin-5 and are processed into mature miRNA duplex by the ribonuclease Dicer [87] (Figure 1c). Interestingly, p53 regulates the processing of pri-miRNA to pre-miRNA by interacting with the Drosha processing complex via the association with DEAD-box RNA helicase p68 (DDX5), thus indirectly inducing the transcription of miR-34a, miR-200c, and miR-17-92 cluster [88]. A study has demonstrated that H_2_O_2_ treatment in endothelial cells decreased the expression of Dicer which in turn downregulated the majority of miRNAs that are normally expressed in cerebromicrovascular endothelial cells [89]. Strikingly, ROS production is also regulated by cellular Dicer levels. A study has shown that dicer knockdown downregulated miRNA expression and decreased the production of ROS in human microvascular endothelial cells [90]. Although this has been investigated in non-cancerous cell models, it would be interesting to analyze whether this phenomenon also exists in cancer cells. Furthermore, NFκB can also regulate miRNA expression indirectly by expressing proteins involved in miRNA processing. A study has shown that NFκB activates the transcription of the miRNA processing inhibitor Lin28, which decreased the let-7 levels rapidly leading to Src-induced cellular transformation [91]. Moreover, ROS not only affect miRNA expression but also modify miRNAs directly through oxidation. A study has shown that upon oxidative modification, miR-184 can target the 3′UTR of antiapoptotic proteins BCL-XL and BCL-W, which are non-native targets of miR-184. Oxidized miR-184 induces apoptosis through downregulating the expression of BCL-XL and BCL-W in the rat heart cell line H9c2 [92]. Altogether, these studies indicate that ROS promote cancer progression through controlling miRNA expression, and the mechanisms involved in the ROS-mediated miRNA expression are not limited. Therefore, more novel mechanisms involved in ROS-dependent miRNA regulation continue to be unraveled in future studies. 

## 4. MiRNAs Regulate ROS Homeostasis

**Figure 2:**
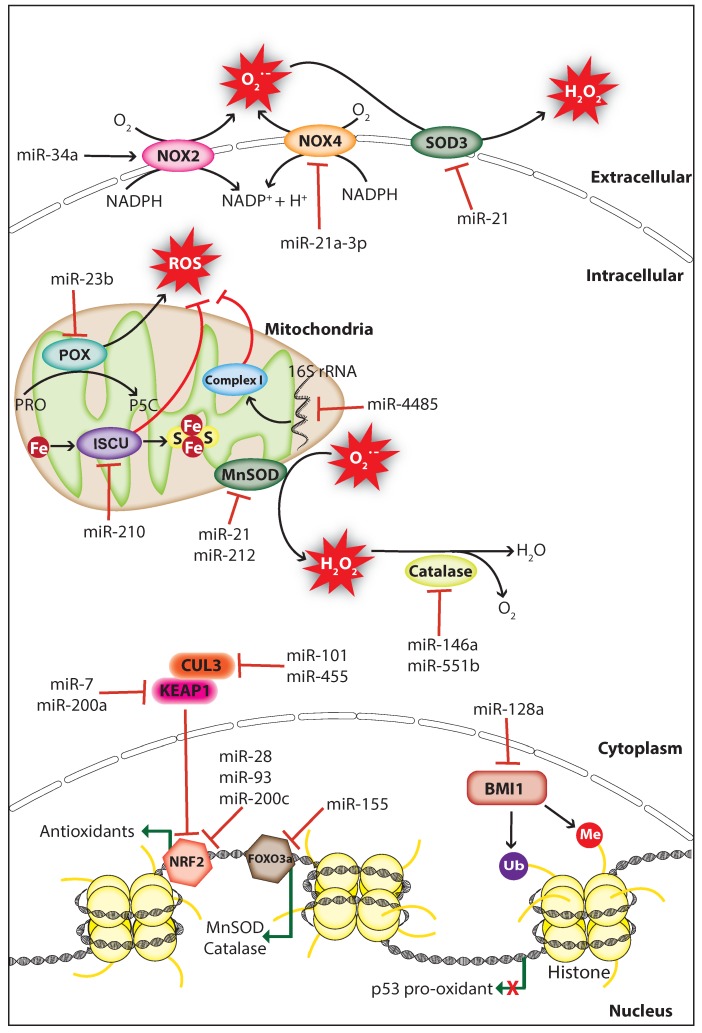
MiRNAs regulate ROS levels in cancer. MiRNAs regulate ROS levels in cancer by inhibiting the expression of ROS producers or antioxidants. MiRNAs decrease ROS levels through inhibiting ROS producers NOX2 (NADPH oxidase 2), NOX4 (NADPH oxidase 4), POX (Proline oxidase), or indirectly by inhibiting the polycomb complex protein BMI1 which repress p53 pro-oxidant expression. ROS levels are elevated by miRNAs through direct or indirect inhibition of antioxidants including catalase, SOD3 (Superoxide dismutase 3), MnSOD (Manganese-dependent superoxide dismutase), and proteins involved in mitochondrial function including mitochondrial complex I (NADH Coenzyme Q reductase) and ISCU (Iron-sulfur cluster assembly enzyme). BMI1, B lymphoma Mo-MLV insertion region 1 homolog; CUL3, cullin-3, Fe, iron; FOXO3a, forkhead box O3; KEAP1, Kelch-like ECH-associated protein 1, NRF2, nuclear factor-erythroid 2-related factor 2; H_2_O_2_, hydrogen peroxide; Me, methyl group; NADPH, nicotinamide adenine dinucleotide phosphate; O_2_^−^, superoxide; PRO, proline; P5C, 1-pyrroline-5-carboxylate; S, sulfur; Ub, ubiquitin (The black arrow indicates upmodulation, the red T arrow indicates inhibition, the green arrow indicates transcription activation, the green arrow with red cross indicates transcription inhibition).

MiRNAs can affect cellular redox homeostasis by regulating the expression of endogenous ROS producers and antioxidants. They usually manipulate ROS levels by directly targeting the genes involved in ROS production or elimination processes (Figure 2). In this section, we discuss how miRNAs control cellular ROS levels in cancer by targeting genes involved in redox homeostasis. 

### 4.1. Regulation of ROS Producer

Studies have shown that miRNAs can affect the expression and function of endogenous ROS producers through functional interactions, thereby controlling cellular ROS production in cancer cells. The membrane-bound enzyme NADPH oxidases (NOXs) produce O_2_^−^ through catalyzing the reduction of O_2_ by transferring an electron from NADPH [93]. The tumor suppressor miR-34a regulates NOX2, the catalytic subunit of NADPH oxidase and overexpression of miR-34a in glioma cells induced apoptosis through NOX2 mediated ROS production [94]. Proline oxidase (POX) is a p53-activated ROS producer whose expression is decreased in human cancer tissues including renal cancer. POX is a direct target of miR-23b, and knockdown of miR-23b promotes ROS production and apoptosis thereby inhibiting kidney tumor growth [95]. Knockdown of dicer in mouse endothelial cells increased the activity of miR-21a-3p targeting NOX4 3′UTR, which resulted in decreased cellular ROS production and endothelial cell tumor formation [96]. These findings indicate that ROS can act as a double-edged sword, thus both overproduction or inhibition of ROS can have a significant effect on cancer progression.

### 4.2. Regulation of Mitochondrial Functions 

Mitochondria are the major site for ROS production, and redox homeostasis in mitochondria is crucial for normal cellular processes. MiRNAs have been shown to affect the ROS production of mitochondria in cancer cells by regulating genes associated with mitochondrial function. The hypoxia-induced miR-210 promotes ROS production by repressing the iron-sulfur cluster assembly enzyme (ISCU) which is essential for the assembly of iron-sulfur (Fe-S) cluster and mitochondria respiratory activity [97]. However, a study suggests that miR-210 mediated ROS accumulation may be due to the repression of other gene targets since the ISCU knockdown in colon cancer cells does not increase ROS levels significantly [98]. A study has shown that miR-128a promotes intracellular ROS levels and cellular senescence in medulloblastoma cells by directly targeting the polycomb complex protein BMI-1 which is involved in the maintenance of mitochondrial activities and redox homeostasis [99]. Surprisingly, a study demonstrated that miRNAs regulate ROS production by targeting non-coding RNAs, during cellular stress miR-4485 translocates to mitochondria and directly targets mitochondrial 16S ribosomal RNA (rRNA), thus modulates mitochondrial function and subsequent ROS accumulation (Figure 2). Importantly, miR-4485 levels are decreased in human breast cancer tissues and overexpression of miR-4485 suppressed breast cancer tumorigenesis in-vitro and in-vivo [100]. These findings strongly suggest that hindrance in mitochondrial metabolism can promote carcinogenesis through ROS accumulation. 

### 4.3. Regulation of Antioxidants

Antioxidant enzymes and non-enzymatic antioxidants mediate the detoxification of ROS to protect cells from oxidative damage. Superoxide dismutase (SOD) is an antioxidant metalloenzyme expressed in both eukaryotes and prokaryotes, which utilizes the metal ions including copper, iron, manganese, and zinc as cofactors to catalyze the dismutation of O_2_^−^ into molecular oxygen (O_2_) and H_2_O_2_. Similarly, catalase is an antioxidant enzyme located mostly in the cytosol and peroxisomes scavenge ROS through catalyzing the conversion of H_2_O_2_ into water (H_2_O) and O_2_ [101]. Several studies have shown that miRNAs can upregulate cellular ROS levels in cancer cells by inhibiting antioxidants including SOD and catalase. The oncomiR miR-21 promotes tumorigenesis through increasing cellular ROS levels by directly targeting the SOD3 or by targeting TNFα that results in MnSOD downregulation (Figure 2) [102]. Furthermore, the miR-212 which is downregulated in human colorectal cancer (CRC) can regulate MnSOD by directly targeting its mRNA, and overexpression of miR-212 inhibited metastasis of CRC cells by suppressing MnSOD expression [103]. In cancer cells, catalase expression is regulated by miR-551b and miR-146a, and inhibition of catalase by these miRNAs promotes ROS accumulation [104,105]. Interestingly, miRNAs can also control the expression of antioxidants indirectly through targeting transcription factors that promote the transcription of antioxidants. For example, K-Ras-induced miR-155 increases ROS levels by directly targeting FOXO3a, a transcription factor that activates the transcription of antioxidants MnSOD and catalase (Figure 2) [106]. These findings suggest that the endogenous expression of endogenous antioxidants is crucial for the prevention of cellular ROS accumulation, which is manipulated by miRNAs in cancer cells to support cancer progression. 

### 4.4. Regulation of NRF2/KEAP1 System

Cellular redox homeostasis is controlled by the nuclear factor-erythroid 2-related factor 2 (NRF2)/ Kelch-like ECH-associated protein 1 (KEAP1) system. NRF2 is a transcriptional factor, which activates the transcription of genes that encode antioxidant enzymes and non-enzymatic antioxidants in response to oxidative stress. Under normal conditions, NRF2 is inactivated by the KEAP1-cullin3 (CUL3) complex, which sequesters NRF2 in the cytoplasm and promotes NRF2 degradation through ubiquitination. During oxidative stress, NRF2 is dissociated from the KEAP1-CUL3 complex caused by the rapid oxidation on cys151 residue of KEAP1 [107]. In cancer, miRNAs can affect the cellular redox homeostasis by targeting genes involved in the NRF2/KEAP1 regulatory system. Overexpression of miR-200c in lung cancer cells increases ROS levels through suppressing the expression of proteins involved in oxidative stress defense including peroxiredoxin 2, NRF2, and Sestrin 1 [108]. A study has shown that miR-28 decreases NRF2 expression by directly targeting its 3′UTR, which increased the colony formation capacity in breast cancer cells [109]. Similarly, miR-93 regulates NRF2 and is associated with breast cancer development [110]. Moreover, a bioinformatic prediction showed that about 85 miRNAs may negatively regulate NRF2 expression by directly targeting its mRNA [111]. miRNAs also regulate NRF2 activity indirectly through targeting its inhibitors KEAP1 and CUL3 (Figure 2). miR-7 and miR-200a target KEAP1 mRNA and decrease its protein expression thereby mediating NRF2 nuclear localization and target gene transcription in neuroblastoma and breast cancer cells, respectively [112,113]. Likewise, miR-101 and miR-455 target CUL3 mRNA, which promotes NRF2 nuclear localization that leads to angiogenesis and oxidative stress protection, respectively [114,115]. Altogether, these studies strongly suggest that cancer cells manipulate ROS levels by controlling miRNA expression to support their survival and promotion.

## 5. The Interplay of ROS and MiRNAs in Cancer 

**Figure 3:**
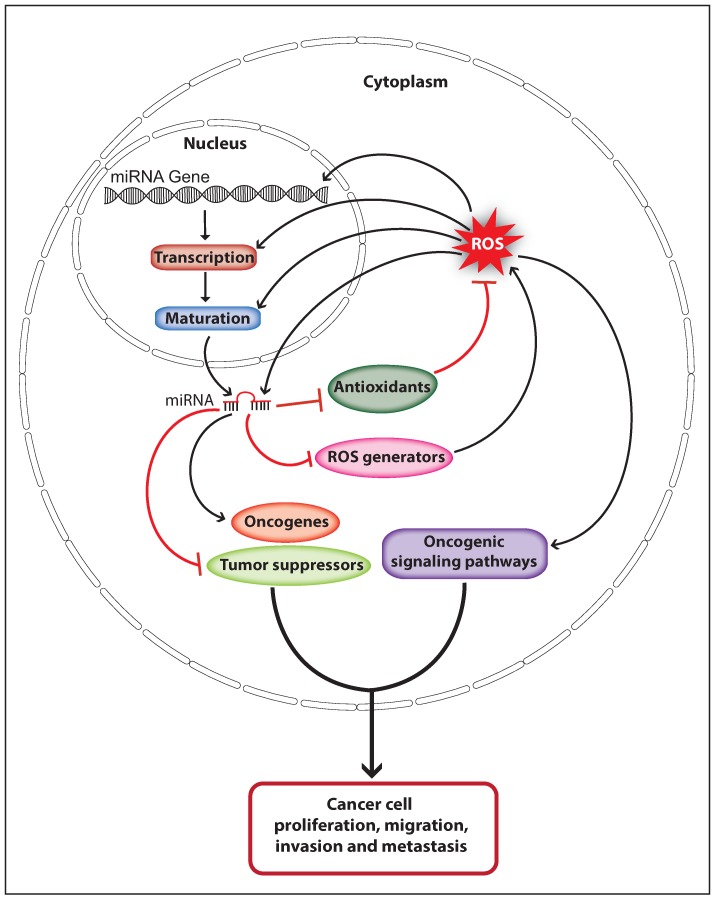
Schematic model illustrates the interplay of ROS and miRNAs in cancer progression. ROS regulate miRNA expression and function by altering miRNA transcription, maturation, or sequences. Dysregulated miRNA expression promotes cancer progression by regulating oncogenes and tumor suppressors. MiRNAs control cellular ROS levels by regulating endogenous antioxidants and ROS generators, which favor cancer development through activating oncogenic signaling pathways (The black arrow indicates upmodulation, the red T arrow indicates inhibition).

Oxidative stress induces DNA damage or mutation that may affect the expression and function of genes associated with the damaged genomic loci, and can eventually cause cancer initiation. ROS may also affect miRNA expression and function directly by causing oxidative damage-induced mutation on miRNA genes and mature miRNA sequences, or indirectly by altering its epigenetic signature or biogenesis pathway. Deregulated miRNA expression caused through genomic deletion, epigenetic silencing, or overexpression can contribute to cancer initiation and progression by controlling oncogenes and tumor suppressor genes. Therefore, ROS can regulate miRNA-mediated carcinogenesis. Elevated ROS production is observed in various cancer types and high cellular ROS can activate oncogenic signaling pathways that support cancer progression. MiRNAs are able to control the cellular ROS levels by targeting genes involved in ROS production and elimination, thus miRNA can control ROS-mediated carcinogenesis. These facts suggest that ROS and miRNAs can function synergistically in the process of cancer development (Figure 3). ROS upregulate the expression of the oncomiR miR-21 and miR-146a through activating NFκB, and these miRNAs can increase cellular ROS levels by downregulating endogenous antioxidants [73,102,105]. Similarly, the miR-210 expression is upregulated through ROS-mediated activation of HIF-1α, and the miR-210 has been shown to increase ROS production by negatively regulating ISCU [97,116]. Interestingly, ROS can also upregulate the expression of the tumor suppressor miR-34 through p53 activation, whereas the miR-34 has been shown to increase ROS production by upregulating the expression of NOX2 [64,94]. These studies strongly suggest that ROS and miRNAs crosstalk in cancer cells to orchestrate the ROS production to activate and promote cancer development. Furthermore, it is of importance to investigate whether the mRNA of genes involved in miRNA expression, ROS production, and detoxification would function as potential competing endogenous RNAs (ceRNAs) which can co-regulate each other’s expression by competing for binding to shared miRNAs [117]. For example, miR-210 can directly target the mRNA of HIF-1α and ISCU, suggesting that HIF-1α and ISCU could function as potential ceRNAs [80,97]. Likewise, miR-21 has been shown to downregulate the expression of antioxidants SOD3 and MnSOD [102]. However, miR-21 targets only the mRNA of SOD3 but not the MnSOD. Therefore, it would be interesting to investigate whether the MnSOD mRNA encompasses a binding site for miR-21 or any other miRNA that can target SOD3. Nevertheless, more studies should be done in this perspective to unravel the complete regulatory network between miRNA and ROS in cancer.

## 6. Challenges in Using Antioxidants for Anti-Cancer Therapy

Since ROS mediate cellular damage and oncogenic mutations, usage of dietary supplement with antioxidants was proposed to prevent or treat cancer. Dietary supplement containing antioxidants such as selenium, vitamin E, and β-carotene was tested to reduce the occurrence of cancer in individuals with a history of cancer. This resulted in a significant decrease in total cancer occurrence and overall mortality. Conversely, studies also show that nutritional supplements of antioxidants may promote cancer incidence and mortality [118,119,120]. Moreover, the usage of antioxidants as additional therapy in cancer treatment failed to show beneficiary effect, supplementing breast and colorectal cancer patients with ascorbate/vitamin C does not improve overall or progression-free survival [121]. Even though antioxidants are often ineffective for cancer prevention/treatment in humans with a high risk of cancer, it was shown that antioxidant treatment might suppress cancer risk in mice with certain genetic modifications. NAC treatment reduces ROS generation, DNA damage, and cancer occurrence in mice deficient of ATM and p53 [122,123]. However, this was not consistent since another study demonstrated that treating mouse models of lung cancer with antioxidants NAC and vitamin E promotes tumor progression and decreases mouse survival [124]. The underlying cause of antioxidants promoting cancer progression may be due to the fact that cancer cells are more susceptible to oxidative stress when compared to normal cells. Therefore, cancer cells depend on endogenous antioxidants including GSH, TRX, NRF2, thioredoxin-like 2, SOD, MnSOD, and glutamate-cysteine ligase to protect them from oxidative stress during cancer development [125,126,127,128,129,130]. In cancer cells, several oncogenes increase NRF2 transcription to promote ROS detoxification and tumorigenesis, whereas the deletion of NRF2 promotes DNA damage and suppresses tumorigenesis in pancreatic cancer cells [131]. In some cancers, ROS levels are suppressed by continuous activation of NRF2 achieved via mutations in NRF2 or its inhibitors KEAP1 that prevents NRF2 translocation from nucleus to cytoplasm [132]. Oxidative stress also limits metastasis by melanoma cells, whereas antioxidant treatment in a mouse model of malignant melanoma promotes the distant metastasis without affecting the growth of primary subcutaneous tumors [133]. Furthermore, cancer cells manage ROS levels by increasing NAPDH generation through accelerating multiple metabolic pathways including the pentose phosphate, folate, and malic enzyme pathway [134,135,136]. These studies suggest that antioxidant treatments are beneficial for cancer progression instead of being detrimental to cancer cells. Importantly, the inconsistent outcome from the clinical trials and experimental mouse models suggest that the application of antioxidants for anti-cancer therapy may not be a promising approach. 

On the other hand, miRNAs are suggested as promising therapeutic agents for cancer treatment. In recent years, several studies proposed many novel miRNA-based cancer therapies that have significantly improved the survival of cancer patients [137]. The application of miRNAs as therapeutic agents has many potential advantages. Basically, miRNAs are highly conserved endogenous small RNA molecules with known sequences which may simplify the process of designing therapeutic agents with less off-target effects. A single miRNA can potentially regulate multiple target genes associated with single or multiple pathways which could be a very efficient way to treat multi-pathway diseases including cancer. For anti-cancer therapy, two miRNA-based strategies are applied. MiRNA replacement therapy is applied to either induce apoptosis or suppress the proliferation of cancer cells by using exogenous tumor suppressor miRNA mimics. MiRNA reduction therapy is applied to inhibit the function of oncogenic miRNAs by using antagomiRs or locked-nucleic acids antisense oligonucleotides (LNAs) [138,139]. To date, there is no miRNA-based drug available for cancer treatment. However, some miRNA drug candidates have entered into the early phase of human clinical trials. These include MesomiR-1, the miRNA mimic of tumor-suppressing miR-16 for treating lung cancer; MRX34, the miRNA mimic of tumor-suppressing miR-34 for treating liver cancer, lymphoma and melanoma; and MRG106, the LNA-modified anti-miR of miR-155 for treating T-cell lymphoma [140]. Although miRNA-based therapy has made progress, still there are some challenges ahead to become an efficient therapeutic approach. The adverse effect is one of the major challenges encountered by this therapy. For example, MRX34 has been withdrawn from entering phase 2 trials due to the serious immune response observed in some patients during phase 1 trials [140]. There are limitations in the efficiency of in-vivo delivery of miRNA mimics and antagomiRs as the oligonucleotides are degraded by the endonucleases in the blood. Understanding the regulatory network of miRNA and ROS production in cancer would further help to develop an alternative effective therapeutic approach to treat cancer. One such approach would be aggravating oxidative stress in cancer cells through miRNA-based therapy that either enhance ROS production or inhibit endogenous antioxidant system. 

## 7. Concluding Remarks

ROS function as a mediator of cellular signaling pathways involved in proliferation, growth, survival, and apoptosis, and the redox homeostasis is actively maintained by endogenous antioxidant systems. Cancer cells manipulate the cellular ROS levels to favor their proliferation, survival, and metastasis. ROS levels are regulated via fine-tuning the expression of ROS producers and scavengers by miRNAs. On the other hand, ROS regulate miRNA expression by altering the activity of proteins involved in miRNA transcription and maturation. The regulatory network of ROS and miRNAs is orchestrated in cancer to promote cancer progression and to cope with oxidative stress. Identification of regulatory crosstalk between miRNA and redox signaling opens up new horizons for using miRNAs as potential therapeutic targets in cancer treatment. However, further understanding of the miRNA-ROS regulatory network is needed for the application of miRNAs to augment ROS-mediated cancer cell death.
